# Immune modulation: the key to combat SARS-CoV-2 induced myocardial injury

**DOI:** 10.3389/fimmu.2025.1561946

**Published:** 2025-05-14

**Authors:** Zhaoqing Li, Luning Qin, Xiaojian Xu, Ruolan Chen, Guoliang Zhang, Banghui Wang, Bing Li, Xian-Ming Chu

**Affiliations:** ^1^ Department of Cardiology, The Affiliated Hospital of Qingdao University, Qingdao, Shandong, China; ^2^ Department of Genetics and Cell Biology, Basic Medical College, Qingdao University, Qingdao, China; ^3^ Department of Cardiology, The Affiliated Cardiovascular Hospital of Qingdao University, Qingdao, China

**Keywords:** myocardial injury, immunity, SARS-CoV-2, COVID-19, therapeutics, cardiovascular

## Abstract

The outbreak of severe acute respiratory syndrome coronavirus 2 (SARS-CoV-2), which caused the Coronavirus disease 2019 (COVID-19) pandemic, has posed significant healthcare challenges. In addition to respiratory complications, it has led to severe damage in other organs, particularly the cardiovascular system. Of which, myocardial injury is increasingly recognized as a most significant complication, contributing to the high mortality. Recent research indicates the pivotal role of immune dysregulation in mediating myocardial injury in patients infected with SARS-CoV-2. In this review, we provide a comprehensive analysis of the immune mechanisms involved in SARS-CoV-2-induced myocardial damage, focusing on the roles of key immune cells and molecules that contribute to this pathological process. Aiming at mitigating the myocardial injury of COVID-19, we review immune-based treatments under evaluation in preclinical and clinical trials. Along with talking about the similarities and differences in myocardial injury resulting from SARS-CoV-2, the Middle East respiratory syndrome coronavirus (MERS-CoV) and the severe acute respiratory syndrome coronavirus (SARS-CoV). This article provides a unique perspective on using past experiences to prevent myocardial injury in the face of ongoing virus mutations.

## 1 Introduction

Coronavirus disease 2019 (COVID-19), a highly contagious respiratory illness caused by the severe acute respiratory syndrome coronavirus 2 (SARS-CoV-2), has spread worldwide rapidly and inflicted tremendous harm on the population (1). Different from the two previous pandemics caused by the Middle East respiratory syndrome coronavirus (MERS-CoV) and the severe acute respiratory syndrome coronavirus (SARS-CoV), SARS-CoV-2 has a higher rate of transmission, is extremely contagious, and exhibits frequent mutations (2, 3). SARS-CoV-2 can affect multiple organs in addition to the respiratory system (4), resulting in a variety of extrapulmonary symptoms (5, 6). Among these, cardiac researchers are mainly concerned about myocardial injury induced by the SARS-CoV-2 (7). Infected patients exhibit a variety of cardiovascular symptoms, including myocarditis, pericarditis, heart failure, and arrhythmias (8). Compelling evidence has indicated a compact-connection between the emergence of myocardial injury and adverse events, which lead to a higher risk of mortality (9). As a result, early identification and intervention to avoid myocardial injury plays a key part in determining the outcome and prognosis of COVID-19 patients.

Currently, the pathophysiological process in myocardial injury caused by SARS-CoV-2 infection may be divided into two categories: direct myocardial injury and indirect myocardial injury (10). Some viewpoints speculate that immune dysfunction may participate in the above process and have an obvious impact (11, 12). Immune intervention is thought to be a treatment strategy for myocardial injury related to SARS-CoV-2. This article describes in detail the immune-related factors that contribute to SARS-CoV-2-induced myocardial injury. It also discusses potential strategies for preventing and treating this damage. We undertake a comparison of the myocardial injury induced by SARS-CoV-2, MERS-CoV, and SARS-CoV. Meanwhile, we provide valuable insights into how to learn from past experience to prevent myocardial injury proactively in light of ongoing virus mutations.

## 2 Excessive inflammation and abnormal immune responses in SARS-CoV-2-induced myocardial injury

The immune system is the first line of defense against virus reproduction and transmission, and is made up of two parts: innate immunity and adaptive immunity (13). During the initial stages of SARS-CoV-2 infection, the pathogen-associated molecular patterns (PAMPs) of SARS-CoV-2 are recognized by pattern recognition receptors (PRRs) of host cells (14). This recognition triggers a series of signaling cascades, ultimately inducing the production of type I and type III interferons (IFNs) along with proinflammatory cytokines and chemokines (15). The IFNs enhance the antiviral state of host cells and limit the replication and spread of the SARS-CoV-2, while cytokines and chemokines recruit innate immune cells, including neutrophils, monocytes, and macrophages, to infection sites (16). Dendritic cells (DCs), a kind of innate immune cell, capture the viral antigen at the site of infection and migrate to secondary lymphoid organs such as lymph nodes (17). DCs present the antigen to T cells, thereby activating T cell-mediated adaptive immune response (18). At the same time, innate immune-derived cytokines such as interleukin-1 (IL-1), interleukin-6 (IL-6), and tumor necrosis factor-α (TNF-α) can serve as warning signals to promote the activation, proliferation and differentiation of T cells and B cells. Virus-specific antibodies from B cells and T cell responses synergistically contain the infection (19). This coordinated response establishes that adaptive immunity requires innate immune priming through both antigen presentation and cytokine signaling (20). However, SARS-CoV-2 employs immune evasion mechanisms that delay innate immune activation (21). This impairment allows uncontrolled viral replication while postponing adaptive immunity initiation (22). If the adaptive immune response is delayed too long, elevated viral loads trigger compensatory hyperactivation of innate immunity, resulting in the production of a large number of cytokines and chemokines (16). The resultant overproduction of inflammatory mediators disrupts the balance between the anti-inflammatory and pro-inflammatory responses (23). A large number of cytokines and chemokines cause cytokine storm, leading to various irreversible tissue damage, including to the lung, heart, and kidney (4). It is worth mentioning that cytokine storm-related hyperinflammatory syndrome is the root cause of many severe COVID-19 symptoms, which is also considered the underlying mechanism of COVID-19-related myocardial injury (24).

**Figure 1:**
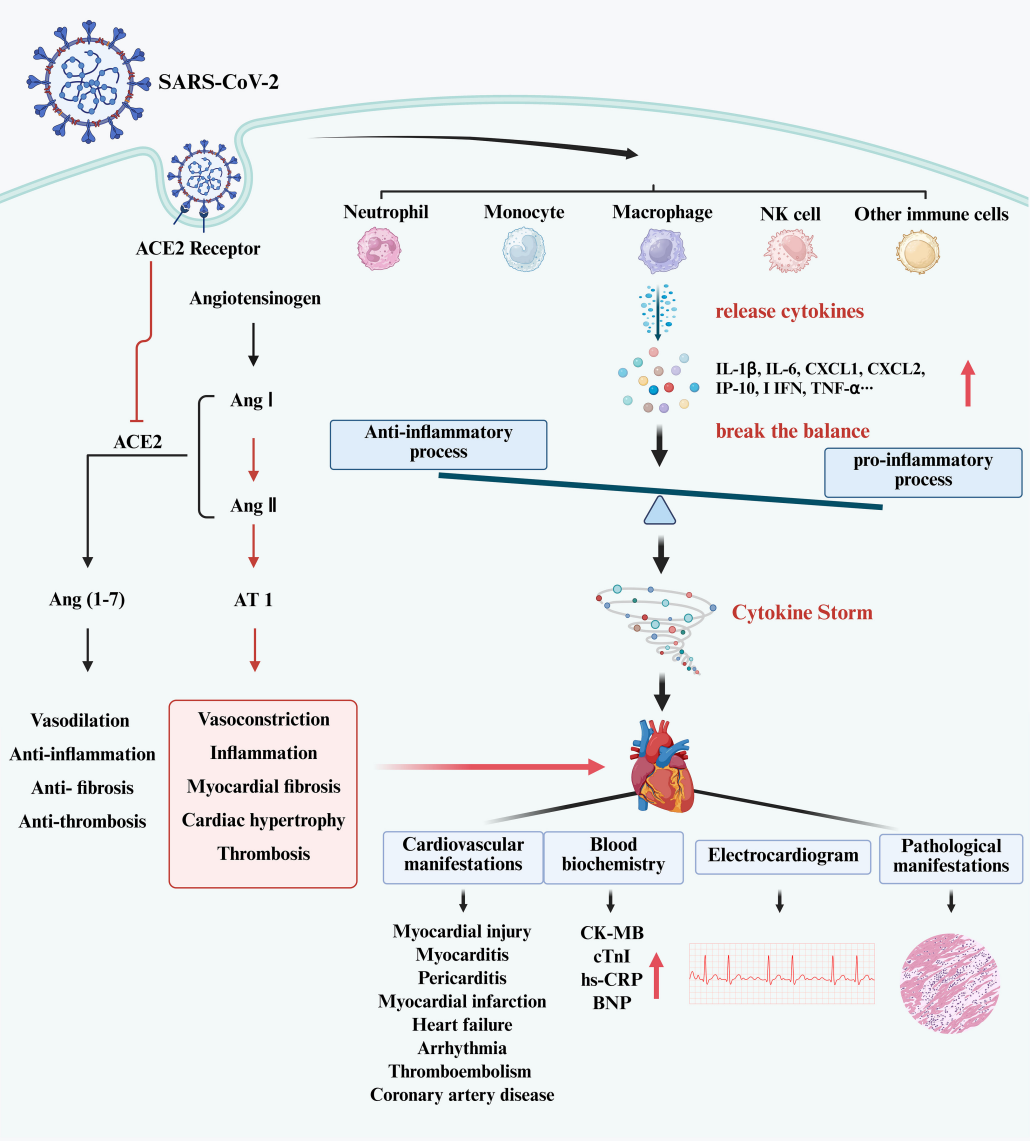
The process of myocardial injury in patients with SARS-CoV-2. (Created in BioRender.com). SARS-CoV-2 infects host cells and activates immune cells, such as neutrophils, monocytes, macrophages, NK cells, T cells, DCs, etc. Those immune cells release cytokines leading to elevated levels of I L-1β, IL-6, CXCL1, CXCL2, I IFN, TNF-α, etc. This breaks the balance between pro-inflammatory and anti-inflammatory processes in the body, produces the cytokine storm, and causes myocardial injury. In clinical practice, myocardial injury may manifest as an increase in CK-MB, cTnI and BNP, with electrocardiographic and pathological changes. In addition, the entry of SARS-CoV-2 into cells has been shown to suppress ACE2 expression. This phenomenon leads to the accumulation of Ang II, resulting in an imbalance between the ACE/Ang II/AT1 axis and the ACE2/Ang1-7/Mas axis. An excessive combination of Ang II and AT1 has the potential to elicit vasoconstriction, inflammation, and myocardial fibrosis. Furthermore, the binding of the S protein of SARS-CoV-2 to the ACE2 receptor will activate the downstream signal cascade, ultimately causing elevated cytokine levels.

Myocardial injury is a common complication of COVID-19. It is an independent risk factor for in-hospital death (9). Infection with SARS-CoV-2 in the host may induce cardiac dysfunction and generate cardiovascular complications (25). In patients with severe and fatal COVID-19, indicators of inflammation and myocardial damage increased dramatically (26, 27). Direct virus infection and the abnormal immune response may be the primary causes of this type of myocardial injury (28–30). Infection with SARS-CoV-2 causes cardiomyocyte dysfunction, inflammation, and cardiac fibrosis, as well as significant aggregation of activated T cells and macrophages (31). The overactive immune response may contribute greatly to the mechanism of myocardial injury. The process of myocardial injury in patients with SARS-CoV-2 has been shown in **Figure 1**.

### 2.1 Immune molecules in SARS-CoV-2-induced myocardial injury

#### 2.1.1 Cytokines——the central mediators of cytokine storm

Cytokines are a form of small-molecule protein released by various kinds of immune cells, which are principally engaged in cellular signal transduction. Cytokines encompass interleukins, interferons, chemokines, tumor necrosis factors and so on (23). A previous retrospective study showed that COVID-19 patients exhibit different degrees of elevated levels of inflammatory factors, including IL-1β, IL-2, IL-6, IL-7, IL-8, and TNF-α. The sudden surge in these pro-inflammatory compounds is known as “cytokine storm” (32). Emerging evidence links COVID-19-associated myocardial injury to cytokine storm-mediated hyperinflammation (33). A clinical study indicates that patients with SARS-CoV-2-induced myocardial injury exhibit characteristic laboratory findings. In addition to elevated levels of high-sensitivity C-reactive protein (hs-CRP), COVID-19 patients with myocardial injury also presented with low lymphocyte count, and high levels of IL-6, IL-8, N-terminal pro-B-type natriuretic peptide (NT-proBNP) and TNF-α (34). The above-mentioned biomarker profile implies a potential pathogenic connection between excessive cytokine release and myocardial injury, particularly given the established association between cardiac involvement and adverse clinical outcomes in COVID-19 (26, 35). Therefore, it is necessary to clarify the key role of cytokines in COVID-19-related myocardial injury and, based on this, inhibit cytokine storm to alleviate myocardial injury caused by SARS-CoV-2.

IL-6 is one of the primary mediators of cytokine storm, which is released during infection (36). IL-6 participates in initiating and amplifying the cytokine storm and coordinating the proinflammatory response of immune cells (37). Infection with SARS-CoV-2 may activate T cells, monocytes, macrophages, DCs, and other immune cells, hence producing IL-6 (38). IL-6 has antiviral activity during viral infection, which might promote inflammation resolution and tissue remodeling (39). However, high levels of IL-6 induce T helper 17 (Th17) cells to produce IL-17, which affect the immune defense system and prolong the duration of viral infection collectively (40).

High levels of IL-6 may be the cause of SARS-CoV-2-induced myocardial injury (41). When researchers exposed rat cardiomyocytes to serum from COVID-19 patients, they discovered a significant expression of acute cardiac inhibition a and persistent arrhythmogenic effects on the cardiomyocytes. Serum levels of cytokines including IL-1, IL-6, TNF-α, and others increased significantly (42). Combinatorial inhibition of IL-6 and TNF-α partially restores the viability and function of cardiomyocytes (33). High levels of IL-6 may be associated with cardiac electrophysiological abnormalities in COVID-19 patients. According to several research, an increase in IL-6 is the primary pathogenic factor in COVID-19-related heart rate to repair the QT interval prolongation (43). In patients with severe COVID-19, the activation of systemic inflammatory response can promote QTc prolongation by increasing IL-6, resulting in cardiac electrical remodeling (44). IL-6 influences myocardial stability by regulating of Na^+^, K^+^ and Ca^2+^ currents (45, 46). This regulatory process ultimately contributes to the occurrence of detrimental cardiovascular events, including arrhythmias associated with COVID-19. Specifically, COVID-19-related heart failure showed a positive correlation with IL-6 (47). Injecting IL-6 into mice during animal studies resulted concentric hypertrophy and cardiac fibrosis, which increased myocardial stiffness (48). These findings reveal that IL-6 may both be a biomarker and a possible therapeutic target in cases of heart failure following SARS-CoV-2 infection. Assessing the levels of IL-6 in the circulatory system can serve as a reliable indicator for predicting the likelihood of mortality in COVID-19 patients. Given the key role of IL-6 in triggering cytokine storm and myocardial injury, targeting IL-6 could be a promising approach to alleviate over-activated immune responses and myocardial injury.

#### 2.1.2 The role of chemokines in myocardial injury caused by SARS-CoV-2

Chemokines are a subset of cytokines characterized by their tiny size. They have the ability to attract immune cells to regions of inflammation or infection, acting as chemical mediators in the recruitment of immune cells during an immune response (49). Upon entering the cell, the virus releases its single-stranded RNA molecules into the cytoplasm. The RNA molecules are then identified by the host’s intracellular pattern recognition receptor. As a result, a sequence of cascade signals is activated, eventually leading to the transcription of proinflammatory cytokines and chemokines (21). In the human-induced pluripotent stem cell-derived cardiomyocytes (hiPSC-CMs) model of SARS-Cov-2 infection, inflammatory cytokines like IL-6, IL-8, C-X-C motif chemokine ligand 1 (CXCL1), C-X-C motif chemokine ligand 2 (CXCL2), and TNF-α are increased (50). Newly generated chemokines exhibit chemotactic properties towards many immune cells implicated in innate immune responses, such as monocytes, macrophages, DCs, and NK cells, among others (51). Chemokine ligand 2 (CCL2), alternatively referred to as monocyte chemoattractant protein-1 (MCP-1), is a member of the chemokine family. This family of molecules has a vital function in attracting leukocytes to areas of infection or injury, which aids in immune defense and tissue repair (52). The C-C chemokine receptor type 2 (CCR2), which binds to the chemokine CCL2, is mostly expressed in monocytes. CCL2 is critical in facilitating the recruitment of monocytes to inflamed regions (53). Once recruited, circulating monocytes can induce the production of tissue factor by releasing cytokines derived from activated platelets and endothelial cells. The aforementioned procedure facilitates the formation of thrombus (54). Suppressing the CCL2/CCR2 axis has been proven to impair the aggregation and adherence of arterial platelets to monocytes, hence mitigating plaque development (55). CCL2 levels were shown to rise progressively in severe COVID-19 patients with high D-dimer levels. This observation implies that CCL2 may be involved in the thrombotic inflammatory processes associated with COVID-19 (52). Yang et al. used the hamster model to show that when SARS-CoV-2 infects cardiomyocytes, it releases CCL2, which attracts monocytes (56). A recent animal study discovered that SARS-CoV-2-induced acute respiratory distress syndrome (ARDS) enhanced cardiac inflammation by enlarging the CCR2+ macrophage subset, potentially leading to cardiomyopathy (57). The evidence presented suggests that CCL2 plays an integral part in the development of cardiovascular disease. Suppressing the CCL2/CCR2 axis may mitigate adverse cardiovascular events in COVID-19 patients by restricting the aggregation of monocytes and macrophages at infection sites.

#### 2.1.3 Impaired interferon and immune evasion in myocardial injury caused by SARS-CoV-2

**Figure 2:**
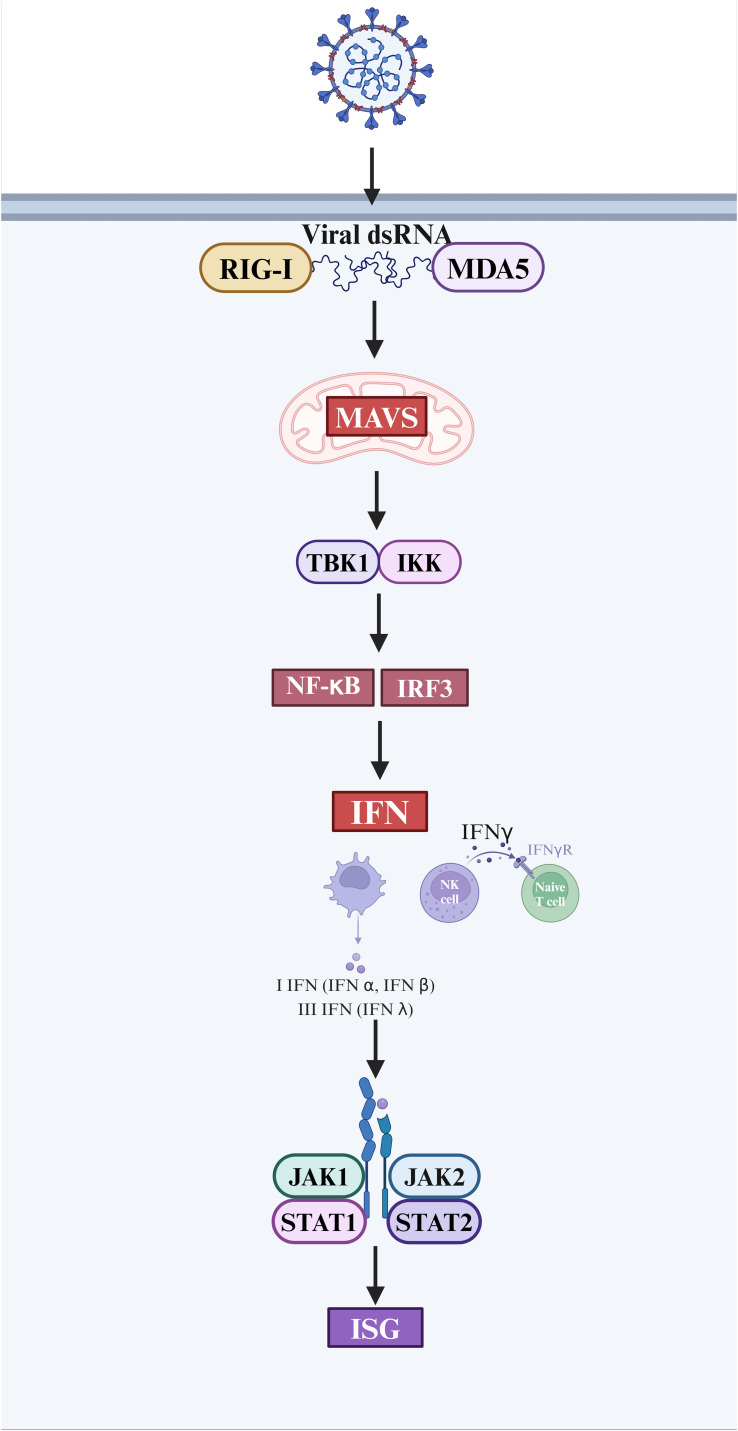
The interferon response and antiviral process after SARS-CoV-2 infection. (Created in BioRender.com).

Interferons are one of the important cytokines in innate immune responses (58). Following viral infection of the host, PRRs identify PAMPs and damage-associated molecular patterns (DAMPs), leading to the synthesis of type I and III interferons and proinflammatory cytokines to trigger antiviral responses (14). Type I interferon is mostly composed of IFN-α and IFN-β (59). The IFN-λ family is a subtype of type I interferon, which is also referred to as type III interferon. Following virus infection, all nucleated cells release substantial levels of type I and III interferon. T cells, NK cells, and macrophages produce type II interferon (IFN-γ) (60). Upon entry into the host cell, the viral double-stranded RNA (dsRNA) is detected by the RIG-I/MDA-5 receptors (61). This recognition triggers a series of antiviral signaling events, facilitated by the interaction between RIG-I/MDA-5 and mitochondrial antiviral signals (MAVS). Following this, the MAVS triggers the activation of Iκ B kinase α/β (IKK) and TBK1/IKK ϵ. Then, these kinases turn on the transcription factors NF- κB and IRF3, which causes genes that code for interferon to be transcribed (62). Type I or Type III interferon interact with certain receptors in order to initiate antiviral defense mechanisms via the JAK-STAT signal transduction pathway. The activation of IFN induces the upregulation of gene expression, specifically the expression of interferon-stimulated genes (ISG). This subsequently leads to the production of antiviral effector protein, conferring antiviral capabilities to the host cells (63, 64) (**Figure 2**).

Type I interferon, such as IFN-α and IFN-β, is essential in fighting against SARS-CoV-2 by effectively regulating the immune response. It is indispensable in regulating viral replication and mitigating the risk of illness exacerbation. Host cells treated with type I interferon significantly inhibit the replication of SARS-CoV (65). However, patients afflicted with COVID-19 frequently exhibit impaired type I interferon response, leading to a protracted elimination of the viral pathogen (66). In the context of influenza infection, the immune response involves the activation of antiviral mechanisms mediated by interferon, which typically precedes the pro-inflammatory response. This temporal sequence seems to enhance host protection while minimizing potential harm to surrounding tissues. But the immunological process described above is not applicable to COVID-19. In hospitalized patients with SARS-CoV-2 infection, we found that the production of IFN- λ and I IFN was reduced or delayed, which could only be produced when a small percentage of patients were in severe condition. Compared to that, pro-inflammatory cytokines, such as TNF-α, IL-6, and IL-8, were observed to be generated prior to the interferon response across all patients (67). During the course of COVID-19, SARS-CoV-2 has the ability to trigger a delayed release of type I interferon during the initial stage of infection. This phenomenon allows the virus to avoid immune system surveillance, leading to its replication within the host’s body and ultimately ending in immunological escape (68). The virus demonstrates the capacity to evade the early innate immune response, specifically the type I interferon response (69), while the activation of the adaptive immunological response is contingent upon the initiation of the innate immune alarm. Consequently, immune escape occurs (70, 71). This reaction attenuates and exacerbates viral replication, leading to excessive activation of Th1 cells. Moreover, the production of INF-γ serves to activate macrophages, triggering the secretion of inflammatory cytokines. This, in turn, leads to a cytokine storm, exacerbating the detrimental effects on the heart.

In the vitro model of manufactured heart tissue, Zhan et al. discovered that the application of IFN-γ may promote a decrease in cardiac contractility and lead to cardiomyocyte dysfunction. The proinflammatory cytokine IFN is linked to an increased risk of cardiac dysfunction. The structural and functional abnormalities generated by IFN are attributed to changes in the balance of pro- and anti-inflammatory cytokines, together with the activation of JAK/STAT signaling pathways. This validates the preceding procedure (72). Furthermore, the investigators conducted a comprehensive examination of the JAK-STAT pathway in primary cardiomyocytes. Their analysis revealed that SARS-CoV-2 has the capacity to selectively affect the proximal constituents of the JAK-STAT pathway. In particular, the virus uses ubiquitin to disrupt the integrity of type I interferon receptors. This makes cells less sensitive to type I interferon (73). Several studies have pointed out that the M protein on the surface of SARS-CoV-2 hinders the generation of type I and III interferons by targeting the RIG-I/MDA-5 signal transduction pathway. Consequently, the disruption weakens the host’s antiviral immune response and facilitates the replication of the virus (62). A group of studies have shown that viruses often use interferon signaling suppression to avoid the body’s natural defenses against viral infections. Targeted therapy that targets immune evasion mechanisms might hinder the virus from replicating in people who have COVID-19, which could lower the risk of serious heart problems.

SARS-CoV-2 is sensitive to IFN treatment, pointing the way for COVID-19 treatment (74). However, several investigations have demonstrated that interferon therapy can increase the expression of host Angiotensin-converting enzyme 2 (ACE2), raising the likelihood that aggravates COVID-19. The study conducted by Busnadiego and colleagues revealed that the use of IFN resulted in an increase in both the transcriptional and cellular expression of ACE2. However, it was shown that the antiviral properties of IFN counteracted the viral infection facilitated by ACE2. This conclusion provides valuable insights for reassessing the therapeutic efficacy of interferon as a pharmaceutical intervention (75).

### 2.2 Immune cells in SARS-CoV-2-induced myocardial injury

#### 2.2.1 Macrophages

Macrophages are one of the prominent subsets of immune cells in the cardiac tissue. It assumes a pivotal function in the pathophysiological progression of cardiovascular disease. The role of macrophage polarization and macrophage-induced cytokine storm in the development of cardiovascular problems generated by SARS-CoV-2 has sparked significant interest. In the cardiac tissues of individuals who died from COVID-19, there was a rise in the density of CD68 macrophage infiltration in the myocardium. There is an indication that macrophages might play an essential part in myocardial damage among those affected by COVID-19 (76). Interestingly, recent research has revealed that the spike (S) protein of SARS-CoV-2 directly binds to macrophages through the S-protein-angiotensin-converting enzyme 2 interaction (77). The infected macrophages can increase the levels of reactive oxygen species and apoptosis in cardiomyocytes by secreting IL-6 and TNF-α, ultimately resulting in cardiotoxicity (78). The phenotypic and functional properties of macrophages exhibit significant variations as monocytes undergo migration to several tissues and subsequent differentiation. In the course of research, M1 and M2 phenotypic macrophages were discovered in COVID-19 patients and healthy persons (79). M1 macrophages are known to release pro-inflammatory cytokines, like IL-6, whereas M2 macrophages are characterized by their secretion of anti-inflammatory cytokines, such as IL-10 (80, 81). Following entering the host cell, SARS-CoV-2 causes a reduction in ACE2 expression on the cellular surface (82). This leads to a substantial accumulation of Ang II, which promotes the transformation of macrophages into the M1 phenotype and triggers the release of pro-inflammatory cytokines from other immune cells. This exacerbates the process of cytokine storm, therefore aggravating cardiovascular damage (83). Therefore, the potential efficacy of mitigating the cytokine storm induced by macrophage activation in the treatment of myocardial damage associated with COVID-19 is worth considering.

#### 2.2.2 Neutrophils

Neutrophils engage in the host immunological response triggered by SARS-CoV-2 infection and have a distinctive function in post-inflammatory damage. The activation of neutrophils and platelets was seen to be significantly heightened in patients infected with COVID-19 (84). The interplay between platelets and cells of the innate immune system initiates the activation of the coagulation cascade, impeding the dissemination of infections throughout the bloodstream. Excessive inflammation and aberrant immune thrombosis, however, may raise the risk of cardiovascular disease. The increased interaction between neutrophils and platelets results in heightened inflammation and aberrant immunological thrombosis, exacerbating the progression of atherosclerosis and heart failure (85–87). The COVID-19 cohort exhibited a notable presence of thrombosis in both the major and minor blood arteries inside the circulatory system. Evidence of neutrophil-platelet aggregation, neutrophil-rich clusters within significant thrombotic formations, and the creation of neutrophil extracellular traps (NETs) was found in myocardial thrombosis in COVID-19 patients, which is a hallmark of neutrophil activation (88). These data imply that alterations in circulating neutrophils are the root cause of myocardial thrombosis in COVID-19 patients. Furthermore, the autopsy findings of COVID-19 patients with myocarditis revealed a substantial presence of neutrophil infiltration. There was a notable increase in the rates of troponin I, maximal creatine kinase, D-dimer, IL-6, and TNF-α during the patients’ hospitalization, indicating a considerable occurrence of cardiac injury (89). Accordingly, it is vital to direct focus towards the significance of neutrophils in cardiac damage and systemic disorders among COVID-19 individuals.

#### 2.2.3 Lymphocytes

Patients hospitalized with SARS-CoV-2 frequently have heightened neutrophil counts and a decrease in lymphocyte numbers, as indicated by laboratory analyses. Lymphopenia, characterized by a notable decrease in the count of CD4+ and CD8+T cells, B cells, and NK cells, is frequently observed in individuals with severe instances of COVID-19 (5, 90, 91). As a consequence, there is a notable elevation in the neutrophil-lymphocyte ratio (NLR). NLR reflects the dynamic between the innate immune response and the adaptive immune response. The presence of NLR has been identified as an autonomous risk factor for the severity and mortality of patients diagnosed with COVID-19. Several studies have shown a positive correlation between the levels of NLR and the severity and progression of COVID-19. There is a positive correlation between elevated NLR and the severity and duration of a certain disease (92–94). In relation to cardiovascular illness, there is a potential association between NLR and several outcomes, including all-cause mortality, coronary heart disease, and heart failure. The rise in NLR is frequently linked to heightened morbidity and death rates in cardiovascular illness, serving as a significant prognosticator for unfavorable cardiovascular outcomes (95–97).

## 3 Immune factors in cardiovascular complications from vaccines

Myocarditis or pericarditis is the prevailing cardiovascular complications subsequent to the SARS-CoV-2 vaccination, primarily affecting individuals of the male gender below the age of 40. This occurrence is particularly prominent following the second dose of mRNA vaccines (98, 99). There is evidence that the BNT162b2 and mRNA-1273 vaccines may increase the risk of myocarditis and pericarditis in many studies (100–102). Typically, individuals tend to experience the onset of fever and chest pain within a time frame of 2–4 days after the second dose of the SARS-CoV-2 vaccine. The laboratory tests revealed heightened concentrations of troponin T and creatine kinase. The most common electrocardiogram (ECG) finding was the elevation of the ST segment. There was typical myocarditis in the patients’ cardiac magnetic resonance (CMR) scans, as shown by the presence of late gadolinium enhancement (LGE) and myocardial edema (103–105). Myocarditis typically manifests in persons who possess predisposing conditions. After follow-up, all the myocardial injuries healed. Researchers observed an amplified T cell response in cases of acute myocarditis that occurred four days after vaccination. Meanwhile, endomyocardial biopsy could identify the infiltration of CD4+ cells within the myocardium (106, 107). This observation implies a potential association between vaccine-induced myocarditis and the autoimmune response. The mechanism underlying vaccine-induced myocarditis remains inconclusive. However, ongoing research efforts persist in this area. There is evidence that S protein may evade the recognition of antibodies in the case of people who got myocarditis after being immunized with SARS-CoV-2. These individuals showed a consistent rise in levels of free S protein and did not bind to spike antibodies (108). The potential mechanism by which S protein may induce myocarditis involves its interaction with ACE2, activation of cardiac pericytes, and induction of endothelial cell dysfunction, leading to the mediation of inflammatory processes. Antibody cross-reaction caused by molecular simulation between autoantigen and S protein encoded in vaccines is also considered to be a possible mechanism of vaccine-associated myocarditis. However, after comparing the sequence homology between SARS-CoV-2 stimulating protein-derived peptides and myocarditis-associated antigens, Marrama et al. found that the frequency of spike-derived peptides similar to myocarditis-related antigens was not significantly enriched (109). The empirical findings do not substantiate the perspective that molecular simulation engenders cross-reaction. In addition, Gill et al. highlighted the distinction between some cases and typical myocarditis in their article, specifically noting the presence of catecholamine-mediated stress cardiomyopathy (110). The emotional and physiological response elicited by the SARS-CoV-2 vaccine has the potential to generate an excessive release of catecholamines, initiating an inflammatory response, which may be the cause of vaccine-induced Takotsubo cardiomyopathy (111). In contrast with the adverse events associated with SARS-CoV-2 infection, the incidence of adverse events resulting from vaccination tends to be lower. Furthermore, it is worth noting that some consequences primarily present in individuals with pre-existing medical conditions. Hence, it is imperative to perform a comprehensive physical assessment before administering vaccinations to individuals afflicted with malignancies, cardio-cerebral blood disorders, and other fundamental ailments. Vaccination remains a highly effective and essential strategy for mitigating the COVID-19 pandemic.

## 4 Genetic susceptibility to SARS-CoV-2-induced myocardial injury

SARS-CoV-2 invades host cells through S protein binding to the host ACE2 receptor. High expression of ACE2 promotes the activation of neutrophils, monocytes/macrophages, NK cells, T-helper-1 (Th 1) cells, Th 2 cells and Th 17 cells to secrete cytokines (112). ACE2 polymorphism may be related to the genetic susceptibility of SARS-CoV-2 (113). Compared to wild-type ACE2, K31R and E37K variants of ACE2 have reduced affinity and the K26R and T92I variants show increased S-protein affinity, which makes the host more susceptible (114). The TT genotype of ACE2 is associated with the severity of COVID-19 (115). ACE2 rs2285666 is greatly associated with both the probability of long-term COVID-19 symptoms and the cumulative incidence of Long COVID (116). Research has shown that variations of ACE2 maybe affect the levels of D-dimer, lactate dehydrogenase (LDH), and CRP. The differences of these biomarkers COVID-19 related give a support for the view that ACE2 rs2285666 may be regarded as a genetic susceptibility marker of COVID19 results (117).

Because of the critical role of genes encoding human leukocyte antigen (HLA) molecules in T cell antigen presentation, HLA has become a major focus of genetic association studies for a variety of infectious and immune-mediated diseases (118). HLA genotypes and related polymorphisms can influence the susceptibility and severity of SARS-CoV-2 infections. Augusto et al. demonstrated a strong and significant association between HLA-B * 15:01 and asymptomatic COVID-19 infection (119). The HLA-C * 01 allele demonstrates a significant association with increased susceptibility to SARS-CoV-2 infection. It serves as a specific ligand for KIR2DL2 and KIR2DL3, which are the receptors that inhibit the activity of NK cells. HLA-C * 01 allele affects the early immune response via its specific interaction with inhibitory NK cell receptors (120). HLA haplotypes may impact the incidence of cytokine storm by interference with immune cell activation. The distinctions among other demographic categories should be taken into account (121). Moreover, variants in cytokine genes, including IL1B, IL1R1, IL1RN, IL6, IL17A, FCGR2A, and TNF may correlate with illness vulnerability and cytokine storm (122). For example, the rs1800629 and rs1800795 variations of proinflammatory cytokines significantly influence the clinical outcomes and systemic inflammatory profiles of COVID-19, elevating TNF-α and IL-6 levels, respectively (123). The effective application of genome-wide association studies (GWASs) and Mendelian randomization can help to identify host genetic variation related to diseases which contribute to explore new mechanisms and therapeutic targets.

## 5 Long-term cardiovascular sequelae: from PASC to chronic dysfunction

Many patients frequently experience a range of symptoms that are challenging to recover after the improvement of acute covid-19 infection. We refer to it as the post-acute sequelae of COVID-19 (PASC), commonly termed “Long COVID.” Long COVID may impact the cardiovascular system and lead to sequelae, including coronary artery disease, arrhythmias, autonomic dysfunctions, thromboembolic events, and myocarditis (124). Certain individuals endure chronic chest pain and dyspnea after an acute infection, potentially attributable to cardiac injury or persistent inflammation (125). Others may exhibit postural orthostatic tachycardia syndrome (POTS), inappropriate sinus tachycardia (IST), and orthostatic hypotension (OH), which may be related to cardiovascular autonomic dysfunction, with symptoms including tachycardia, orthostatic intolerance, fatigue, and cognitive impairment (126). Thrombotic events described in the context of coronavirus pneumonia are multifactorial and may be associated with platelet activation, leukocyte recruitment, and excessive inflammatory response due to endothelial dysfunction (127). Of note, adverse cardiovascular outcomes may occur in people with no previous history of cardiovascular disease, even in mild or asymptomatic patients. At present, the treatment strategy is limited to symptomatic treatment. Therefore, the establishment of predictive models based on multi-omics technology to develop multi-target intervention schemes for endothelial repair, neuroimmune regulation and coagulation homeostasis is expected to become the key direction of translational medicine research in the future.

## 6 Strategies for preventing and treating myocardial injury caused by SARS-CoV-2

### 6.1 Immunomodulatory therapy

SARS-CoV-2 commonly induces damage to several organs beyond the respiratory system, such as the heart. The multi-organ injury observed in COVID-19 patients is a result of the combination of cytokine storm and host immune system dysregulation, ultimately resulting in deteriorated clinical outcomes. Therefore, employing immunomodulatory therapy targeting cytokine storm triggered by excessive inflammatory responses may be beneficial to improving patients’ outcomes.

In COVID-19 patients, elevated levels of IL-6 are crucial in the occurrence of cytokine storm, QT syndrome, and Torsades de Pointes (45, 128). Higher levels of IL-6 are related to severe COVID-19 and adverse prognosis (129). The study has found that using anti-IL-6 receptor monoclonal antibody tocilizumab in COVID-19 patients reduced the risk of inflammation-driven arrhythmias (128). So, using IL-6 inhibitors might ultimately diminish the detrimental consequences of elevated IL-6 levels and also protect the heart. In a prospective analysis conducted on clinical trials including patients who were hospitalized with COVID-19, administration of IL-6 antagonists showed a reduction in 28-day all-cause mortality in comparison to traditional treatment or placebo (130). Tocilizumab, a humanized monoclonal antibody that targets to the IL-6 receptor, has the capability to suppress the physiological activity of IL-6 effectively (131). Treatment with tocilizumab in COVID-19 patients led to a decrease in mortality, lower rates of admission to the intensive care unit (ICU), and reduced reliance on mechanical ventilation compared to patients who did not receive tocilizumab medication (132, 133). Particularly, researchers have found that concurrently administering corticosteroids and tocilizumab enhances clinical benefits, thereby establishing it as a potentially safe and advantageous therapeutic approach (134). In addition to tocilizumab, various additional monoclonal antibodies (mAbs) that have the potential to inhibit the physiological impacts of IL-6 are being evaluated in clinical studies for the treatment of COVID-19, including sarilumab, siltuximab, sirukumab (135, 136).

Cenicriviroc (CVC) functions as an antagonist for both C-C chemokine receptor type 5 (CCR5) and C-C chemokine receptor type 2 (CCR2). Cells within atherosclerotic plaques express both CCR5 and its corresponding ligands (137). CVC demonstrates its anti-inflammatory and immunomodulatory characteristics through the antagonism of CCR2 and CCR5, making it a potentially effective treatment option for myocardial infarction. Research findings have indicated that CVC can impede the replication of the SARS-Cov-2 virus (138). The intervention exhibits the capacity to mitigate the occurrence of respiratory and cardiovascular system dysfunction commonly linked to COVID-19 (139). Current clinical trials are examining the effectiveness of CVC, whether used in conjunction with routine care or in combination with other pharmaceutical agents (140). The purpose of these trials is to utilize the anti-inflammatory properties of CVC to improve the clinical advancement of COVID-19 and mitigate the occurrence of comorbidities.

### 6.2 Cell -based therapy

Mesenchymal stem cells (MSCs) are a type of stem cell with the ability of self-renewal and multi-lineage differentiation. MSCs perform diverse functions in reducing inflammation, preventing fibrosis, regulating the immune system, and facilitating tissue regeneration (141). Within the realm of cardiac regeneration, MSCs have been shown to enhance cardiac function through various mechanisms, including immune response regulation, promotion of tissue perfusion, inhibition of fibrosis, and stimulation of cardiomyocyte proliferation (142, 143). Multiple groups of clinical trials have found that the application of MSCs can effectively ameliorate the prognosis of moderate and severe COVID-19 patients, as well as increase the survival rate of COVID-19 patients with ARDS (144, 145). Due to the absence of ACE2 and TMPRSS2 expression, MSCs are less susceptible to SARS-CoV-2 infection (146). Thus, MSCs could serve as a potent therapeutic approach for preventing or treating SARS-CoV-2-induced cardiac injury.

The beneficial effects of MSCs are manifold. MSCs regulate immune cell subsets by secreting paracrine substances, which help coordinate the immune response. Research has shown that intravenous infusion of MSCs can regulate B cell subsets and boost CD28 expression on costimulatory T cells (147). Also, MSCs have the potential to alleviate SARS-CoV-2-related cytokine storm (148). Preclinical models of ARDS have indicated that MSCs exert inflammation suppression effects on host tissues through the release of IL-4, IL-10, transforming growth factor β (TGF-β) and prostaglandin E2 (149). Infusion of MSCs in severe and critically ill COVID-19 patients resulted in a considerable reduction in levels of CRP, pro-inflammatory cytokines, and NETs, as evidenced by clinical trials (147). CPR serves as a biomarker for myocardial damage (150). It is worth noting that patients with elevated IL-6 levels have better infusion effects of human umbilical cord-derived mesenchymal stem cells (hUC-MSCs), as seen by a significant reduction in IL-6 levels and a substantial increase in the oxygenation index (145). This suggests that the inflammatory environment may augment the immune regulatory properties of MSCs. On top of that, MSCs treatment also demonstrates a commendable capacity to stimulate tissue differentiation and regeneration. MSCs migrate to areas of injury and release abundant amounts of growth factors, prompting tissue regeneration and diminishing cellular demise (151). At present, studies in humans have confirmed the safety and efficacy of MSCs. Several clinical trials have verified the effectiveness of intravenous infusion of hUC-MSCs in patients with moderate and severe COVID-19, and it has a beneficial impact on patient-related sequelae after infection (152–154). The enduring safety and efficacy of MSCs therapy has been confirmed and are not associated with serious adverse events (155, 156). Autologous stem cells, especially patient-derived ones, pose no danger of immunological rejection (157).

Cardiosphere-derived cells (CDCs) are stem cells originated from heart tissue (158). CDCs, like MSCs, actively contribute to cardiac repair and point out greater myocardial repair potential than MSCs. The beneficial effects of CDCs in promoting cardiomyocyte regeneration, stimulating angiogenesis, inhibiting inflammation and myocardial fibrosis, enhancing cardiac function, and regulating immunity have been confirmed by research (159–162). Several sets of clinical trials have shown the therapeutic efficacy of CDCs in treating various conditions such as myocardial infarction, heart failure with reduced and preserved ejection fraction, non-ischemic cardiomyopathy, Duchenne muscular dystrophy, and others (161, 163–166). CDCs may have the potential to induce the transformation of M1-like macrophages(pro-inflammatory) into M2-type macrophages (anti-inflammatory), and enhance the ability of macrophages to clear cell debris (167, 168). Due to their capacity to suppress excessive inflammation and facilitate the restoration of myocardium, CDCs could potentially be beneficial for COVID-19 patients suffering myocardial injury (169). Clinical study data indicates that intravenous allogeneic CDCs (CAP-1002) is safe in individuals with severe COVID-19. After administration of CAP-1002, the levels of pro-inflammatory biomarkers were reduced in the majority of patients. Additionally, increased levels of cardiac troponin I and D-dimer were dramatically decreased. And the clinical condition of patients showed improvement (170).

### 6.3 Cell-free therapy based on exosomes

Exosomes are a specific type of nanoparticles with a diameter ranging from 40 to 150 nanometers. Cell-free therapy based on exosomes has shown promise in treating various cardiovascular diseases such as myocardial infarction, myocardial ischemia-reperfusion injury, inflammation of myocardium, and ventricular remodeling (171–173). The therapeutic benefits of MSCs are primarily attributed to exosomes (174). Extracellular vesicles, derived from MSCs, possess comprehensive immunomodulatory and regenerative properties (175). Despite several advantages of MSC-based cell therapy, its potential for causing tumors, the risk of pulmonary embolism, the low *in vivo* survival rate, and challenges with storage present hurdles. As a result, exosome therapy emerges as a promising option (176). Exosomes exhibit lower immunogenicity in comparison to MSCs, hence diminishing the likelihood of thrombosis and the incidence of adverse cardiovascular events (177). Besides, exosomes exhibit robust sustainability and stability within the body, enabling them to effectively and enduringly mitigate inflammation and modulate the immune response, consequently suppressing the onset of cytokine storm (175). As a kind of nanoparticles, exosomes possess the ability to cross the blood-brain barrier, circumventing the risk of pulmonary embolism, which is associated with MSC transplantation. Significantly, exosomes circumvent the potential hazard of tumor development caused by MSCs (141). Exosomes consist of lipids, proteins, mRNA, LncRNA, microRNA, and various other bioactive compounds. Studies revealed that non-coding RNAs contained in exosomes play a pivotal role in cardiac protection (178). The efficacy and security of exosome-based cell-free treatments for patients with COVID-19 have been validated (142). Similarly, extracellular vesicles derived from CDCs are the key mediators of their therapeutic effects (164, 179). In pig models of acute myocardial infarction, intramyocardial injection of CDCs-exosomes significantly reduced cardiac remodeling and enhanced cardiac functions (180). The study discovered that extracellular vesicles derived from CDCs may be involved in regulating the IL-6/IL-6R axis and suppressing the impacts of diseases mediated by inflammation (181). Currently, exosome treatment is primarily applied by either aerosol inhalation or intravenous injection (182). The precise targeting of the heart is a subject of ongoing research, and its potential for transformation is a topic that warrants further discussion.

### 6.4 Cardioprotective agents

The invasion of SARS-CoV-2 can result in severe injury to myocardial tissue, necessitating the creation of a novel medication to counteract the myocardial injury induced by SARS-CoV-2. However, the process of developing and implementing novel drugs requires a certain amount of time. Considering the unpredictability and urgency of the novel coronavirus, looking for cardioprotective agents with antiviral effects among existing clinical drugs is a reliable option. Statins are frequently employed in clinical settings as lipid-lowering medications. It serves a crucial function in the regulation of blood lipid abnormalities and cardioprotective therapy (183). Different observational research has shown that the use of statins diminishes mortality and improves outcomes in COVID-19 patients (184, 185). As a possible candidate for adjuvant treatment of COVID-19 patients with myocardial injury, statins have multiple effects. Research analysis speculates that statins may impede the entry of SARS-CoV-2 into host cells as well as hinder the replication and proliferation of the virus *in vivo* (186, 187). Secondly, statins may have a capacity to reduce the excessive level of pro-inflammatory cytokines and regulate immune responses. A meta-analysis showed that statins have the ability to decrease levels of IL-6 and CRP (188). It exhibits a suppressive effect on cytokine storm and macrophage activation syndrome, which is triggered by raised levels of IL-6. Moreover, statins exhibit remarkable anti-fibrotic potential and accelerate the apoptosis process of fibroblasts against complications induced by SARS-CoV-2 infection (189). In brief, because of its antiviral, anti-inflammatory, anti-fibrotic, immunomodulatory, and cardioprotective properties, statins may be a viable treatment choice for COVID-19 patients with cardiovascular comorbidities. Especially for patients with myocardial damage or dyslipidemia, statins can be used as an auxiliary treatment.

### 6.5 Phytochemicals

Prior reports indicated that phytochemicals, such as alkaloids, flavonoids, and polyphenols, demonstrated antiviral properties on the MERS-CoV and the SARS-CoV (190). Resveratrol (RES), a phenolic molecule, exhibits inhibitory effects against multiple respiratory viruses, such as influenza virus, MERS-CoV, SARS-CoV, and respiratory syncytial virus (191). RES can drastically counter MERS-CoV infection and enhance the survival of virus-infected cells (192). Similarly, *in vitro* experiments show that RES can effectively inhibit the replication of SARS-CoV-2, manifested as reduced virus titer and cytotoxicity (193). In the Vero cell model infected with SARS-CoV-2, the cells treated with RES after infection showed a remarkable inhibition rate of 98% against SARS-CoV-2. After incubating the cells with the virus and RES for 1 hour, followed by the removal of RES interference and subsequent culture for a further 48 hours, the inhibition rate remained approximately 64%. The findings point out that RES exhibits a potent inhibitory effect on the replication of SARS-CoV-2 and can impede viral entry into cells (194). RES can activate the immune system, downregulate the production of pro-inflammatory cytokines, and inhibit cytokine storms. It affects T cells, DCs, and macrophages to control immune responses and minimize tissue and organ impairment (195, 196). A randomized, double-blind, placebo-controlled trial showed that RES can decrease the occurrence of hospitalizations, emergency department visits, and pneumonia in outpatients with mild COVID-19 without generating significant adverse events (197). Furthermore, RES is identified as a cardioprotective drug, which means it can alleviate the cardiotoxicity associated with chloroquine/hydroxychloroquine treatment in SARS-CoV-2 patients (198). Therefore, whether to inhibit viral replication during the initial phase of infection or to reduce systemic inflammation-induced tissue damage and its cardioprotective effects during the later phases, RES seems like a good candidate.

### 6.6 Traditional Chinese medicine

**Figure 3:**
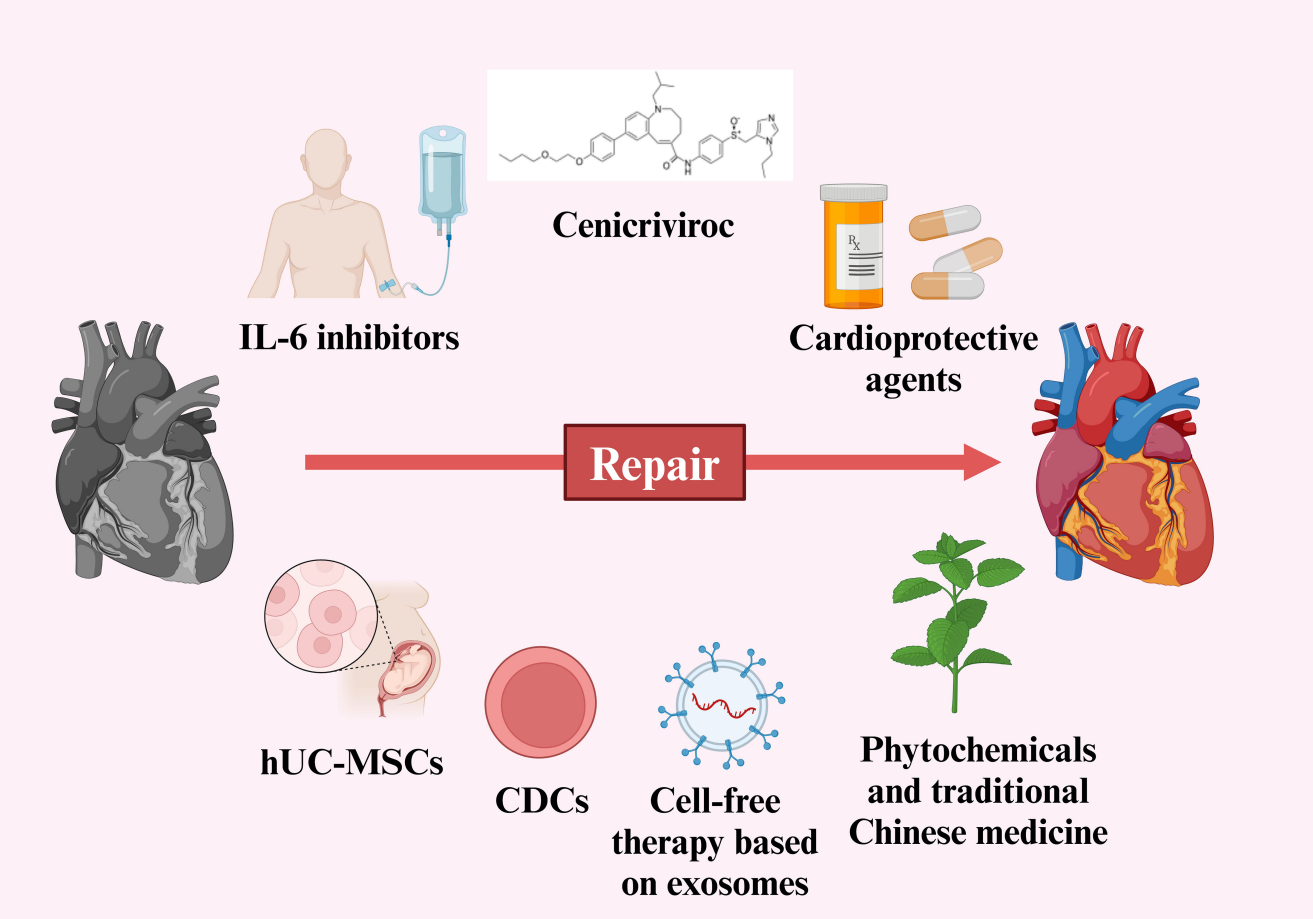
Strategies for preventing and treating myocardial injury caused by SARS-CoV-2. (Created in BioRender.com).

Traditional Chinese medicine (TCM) has been used in previous viral infections, such as SARS-CoV, MERS-CoV and influenza virus (199). After the outbreak of the SARS-CoV-2, the Chinese government quickly adopted a series of prevention and treatment measures, actively promoted the application of TCM, and created three Chinese patent medicines (Jinhua Qinggan Granules, Lianhua Qingwen Capsule and Xuebijing Injection) and three Chinese medicine prescriptions (Qingfei Paidu Decoction, Huashi Paidu Recipe and Xuanfeibaidu Recipe (200). Data from randomized controlled studies of ‘three formulas and three medicines’ indicated that TCM is safe and can mitigate the symptoms of heart damage (201). Whether it is for initial prevention or as an adjuvant treatment for acute myocardial injury induced by SARS-CoV-2, especially for the rehabilitation treatment of post-COVID-19 condition, TCM is a viable option. However, it is necessary to dialectically view the specific conditions of each patient and give a reasonable treatment plan. Strategies for preventing and treating myocardial injury caused by SARS-CoV-2 has been shown in **Figure 3**.

## 7 Discussion and future perspective

SARS-CoV-2, SARS-CoV, and MERS-CoV are all classified as coronaviruses, which primarily attack the respiratory system of humans and lead to a widespread pandemic (202). Prior studies indicated that infection with SARS-CoV-2, SARS-CoV, and MERS-CoV can impact the human immune system and result in multiple systemic impairments (203). SARS-CoV has the ability to invade alveolar epithelial cells and immune cells; however, it can only replicate in epithelial cells. So its impact on the immune system is indirect (204). The distinction of MERS-CoV is its ability to directly invade and reproduce within a range of immune cells, including macrophages, T cells and DCs. Infected macrophages and DCs secrete pro-inflammatory cytokines and chemokines, leading to inflammation and tissue damage (205). SARS-CoV-2 has a comparable effect on the immune system as the previous two viruses. Acute infection with SARS-CoV-2 results in widespread reductions in various types of immune cells, such as T cells, NK cells, monocytes, and DCs. Following infection with SARS-CoV-2, there is a notable occurrence of immunological dysfunction and cytokine storm. The condition of high inflammation and abnormal immune processes ultimately results in the destruction of multiple tissue and organs (206). Similar to SARS-CoV-2, patients infected with MERS-CoV and SARS-CoV were also complicated with cardiovascular diseases (207, 208). However, there is little research on cardiovascular diseases related to SARS-CoV and MERS-CoV infection. The studies primarily focus on clinical case reports and lack the in-depth exploration and summary of relevant mechanisms (208). Consequently, during the SARS-CoV-2 pandemic, there exists a poor awareness of the prevention and management of myocardial injury caused by coronavirus infection. Therefore, in spite of the last coronavirus pandemic has passed, it is essential to explore and summarize the underlying mechanisms of myocardial injury induced by COVID-19. This exploration aims to deepen the comprehension of myocardial injury due to SARS-CoV-2 as well as provide strategies for subsequent prevention and therapy.

It is inevitable to create novel medicines because there are no new therapeutic drugs for myocardial injury in patients with COVID-19 currently. However, the process of researching and developing novel medications and conducting clinical trials requires a certain amount of time. The existing cardioprotective agents with antiviral and immunomodulatory properties may be an appropriate choice. In recent years, treatments based on MSCs have been extensively studied in many fields (148). MSCs have qualities that reduce inflammation, fight against viruses, modulate the immune system, and facilitate the healing of myocardial damage. Clinical trials utilizing MSCs treatment have been carried out in patients with COVID-19. Considering the regular clinical application of MSCs by atomization or intravenous infusion, there exists a requirement to improve the effectiveness of MSCs in treating heart disease. By integrating stem cells with nanotechnology, combination therapies and additional techniques enhance the delivery efficiency and therapeutic efficacy of MSCs (209, 210). Although, cell-free therapies based on exosomes demonstrate unique advantages by avoiding the immunogenicity and thrombotic risks inherent to stem cells. The clinical translation has faced multifaceted challenges. Globally registered clinical trials investigating exosome therapies remain predominantly in Phase II/III development, with a marked predominance of hUC-MSCs as the cellular source. Notably, the development of embryonic stem cell-derived exosomes remains strictly constrained, primarily stemming from persistent ethical controversies (e.g., legal ambiguities regarding embryonic material procurement) and regulatory deficiencies in quality control standardization.

Given the crucial role of cytokine storm in COVID-19-associated myocardial injury, targeted suppression of SARS-CoV-2-induced inflammatory responses may represent an effective therapeutic strategy. The use of IL-6 inhibitors (e.g., tocilizumab), IL-1 inhibitors (e.g., anakinra and canakinumab), NLRP3 inflammasome inhibitors (e.g., colchicine), and JAK inhibitors (e.g., baricitinib) in COVID-19 patients has shown promising results in suppressing inflammation and reducing clinical progression (133, 211, 212). In particular, the anti-thrombotic effect is observed when targeting the NLRP3/IL-1β axis through canakinumab or colchicine administration. Currently, anakinra has been approved for treating hypoxemic COVID-19 patients exhibiting early signs of hyperinflammation, based on its established safety profile and therapeutic efficacy. The non-selective NLRP3 inhibitor colchicine has demonstrated favorable effects in reducing hospitalization and mortality rates among COVID-19 outpatients, despite lacking formal regulatory approval for this indication (213). It is essential to recognize that since excessive immune activation involves the synergistic actions of multiple proinflammatory cytokines, targeting the single inflammatory factor may not be sufficient to suppress excessive inflammation and improve clinical outcomes (214). Therefore, there is still a need to explore specific myocardial injury biomarkers in COVID-19 patients and develop multi-targeted therapy to address the complex cytokine network dysregulation.

At present, the assessment of myocardial injury mainly depends on cardiac troponin I (cTnI) or high-sensitivity cardiac troponin I (hs-cTnI), with increased inflammatory biomarkers (215). Since the elevation of troponin involves both ischemic and non-ischemic causes, there is a need to find more readily available specific and sensitive markers of myocardial injury (216). Non-coding RNA circulating in the blood may be a good choice. Garg et al. assessed changes in circulating cardiovascular miRNA, and the upregulation of miR-21, miR-155, miR-208a and miR-499 in COVID-19 survivors may be predictors of chronic myocardial injury and inflammation. In particular, myocardial-specific miR-208a, and miR-499 showed higher elevations than troponin (217). This provides possible predictive information for the assessment of SARS-CoV-2-related myocardial injury. RNA biomarkers could be useful in the current COVID-19 situation. Although studies put the immune dysregulation into the potential mechanism underlying myocardial injury, there has been a blank in biomarkers between immune factors and myocardial injury. A research study aimed to assess the relationship between myocardial injury and immunologic profiling found that white blood cell count, neutrophil count, types of lymphocyte count (CD3+, CD4+, CD8+, CD19+, CD16+, CD56+), hs-CRP, and procalcitonin had independent correlations with myocardial injury in COVID-19 patients. The elevated indicators above all may give a clue for considering myocardial injury in patients infected with virus (218). The exploration of novel biomarkers for myocardial injury in COVID-19 patients is a direction that warrants future consideration. This provides the possibility for timely identification of myocardial injury and precise targeted treatment. The levels of some biomarkers can be influenced by a variety of factors, including infection and hypoxia. Therefore, the diagnosis of myocardial injury after COVID-19 infection should not only rely on biomarkers but also consider all relevant clinical parameters. Zhong et al. discovered that a decline in myocardial computed tomography (CT) value indicates the presence of myocardial damage. Chest CT is employed to evaluate pulmonary lesions as well as heart morphology and myocardial tissue characteristics in individuals diagnosed with COVID-19. This utilization aims to enhance the clinical utility of chest CT in cardiovascular diseases and furnish patients with additional valuable information (219). Moreover, CMR can also serve as a supplementary diagnostic tool (220). Combined with inflammatory markers, a variety of myocardial injury markers and imaging examination to assess myocardial injury from multiple perspectives, to provide more reliable support for the diagnosis of myocardial injury.

Since the COVID-19 pandemic, various variants of SARS-CoV-2 and their respective branch subtypes have emerged. These include Alpha, Delta, and Omicron variants, as well as their subtypes, such as the Omicron XBB, BA.2.86 and JN.1 variants (221, 222). These subvariants showed higher immune escape ability. Compared with the early original strain, the Omicron mutant strain is the most heavily modified strain among the numerous SARS-CoV-2 variants that have arisen during the COVID-19 pandemic (223). During the surge in Omicron variants, hospitalized COVID-19 patients exhibited a range of myocardial injury manifestations. As previously mentioned, there exists a strong association between severe myocardial injury and higher rates of morbidity and mortality (224). Therefore, it is of utmost significance to recognize the occurrence of myocardial injury in hospitalized individuals infected with SARS-CoV-2 as early as possible, hence facilitating the categorization of COVID-19 patients into risk strata. This enables the selection of appropriate clinical interventions and subsequent treatment strategies for patients (225).

Despite the conclusion of the COVID-19 pandemic, the coronavirus persists and continues to mutate, perhaps leading to another pandemic in the future. We explore the immunological mechanism of SARS-CoV-2-induced myocardial injury, in order to put forward feasible prevention and treatment measures for patients with COVID-19-complicated myocardial injury, so as to strengthen the preparation for the future reinfection wave of SARS-CoV-2 and its variants.

## Glossary

SARS-CoV-2: Severe acute respiratory syndrome coronavirus 2
COVID-19: Coronavirus disease 2019
MERS-CoV: Middle East respiratory syndrome coronavirus
SARS-CoV: Severe acute respiratory syndrome coronavirus
PAMPs: Pathogen-associated molecular patterns
PRRs: Pattern recognition receptors
IFN: Interferon
DCs: Dendritic cell
IL: Interleukin
TNF-α: Tumor necrosis factor-α
ACE2: Angiotensin-converting enzyme 2
CRP: C-reactive protein
hs-CRP: High-sensitivity C-reactive protein
NT-proBNP: N-terminal pro-B-type natriuretic peptide
Th17: T helper 17
hiPSC-CMs: Human-induced pluripotent stem cell-derived cardiomyocytes
CCL2: Chemokine ligand 2
MCP-1: Monocyte chemoattractant protein-1
NK cells: Natural killer cells
CXCL1: C-X-C motif chemokine ligand 1
CXCL2: C-X-C motif chemokine ligand 2
Ang I: Angiotensin I
AT1: Angiotensin II Receptor Type 1
IP-10: Interferon-gamma-inducible protein
I IFN: Type I interferon
CK-MB: Creatine kinase isoenzymes
cTnI: Cardiac troponin I
BNP: Brain Natriuretic Peptide
DAMPs: Damage-associated molecular patterns
dsRNA: Double-stranded RNA
RIG-I: Retinoic acid-inducible gene-I
MDA-5: Melanoma differentiation related gene 5
MAVS: Mitochondrial antiviral signals
TBK1: TANK-binding kinase 1
IKK ϵ: Inhibitor-kappa B kinase
NF- κB: Nuclear factor kappa-B
IRF3: Interferon regulatory Factor 3
ISG: Interferon-stimulated genes
JAK: Janus Kinase
STAT: Signal transducer and activator of transcription
NETs: Neutrophil extracellular traps
NLR: Neutrophil-lymphocyte ratio
ECG: Electrocardiogram
CMR LGE: Cardiac magnetic resonance Late gadolinium enhancement
NK cells: Natural killer cells
Th 1 cells: T-helper-1 cells
LDH: Lactate dehydrogenase
HLA: Human leukocyte antigen
GWASs: Genome-wide association studies
PASC: Post-acute sequelae of COVID-19
POTS: Postural orthostatic tachycardia syndrome
IST: Inappropriate sinus tachycardia
OH: Orthostatic hypotension
ICU: Intensive care unit
S protein: Spike protein
mAbs: Monoclonal antibodies
CVC CCR2: Cenicriviroc C-C chemokine receptor type 2
CCR5: C-C chemokine receptor type 5
MSCs: Mesenchymal stem cells
ARDS: Acute respiratory distress syndrome
UC-MSCs: Humumbilical cord-derived mesenchymal stem cells
TGF-β: Transforming growth factor β
CDCs: Cardiosphere-derived cells
CAP-1002: Intravenous allogeneic CDCs
RES: Resveratrol
TCM: Traditional Chinese medicine
NLRP3: NOD-like receptor thermal protein domain associated protein 3
hs-cTnI: High-sensitivity cardiac troponin I
CT: Computed tomography
